# Identification of a Novel *SLC18A2* Splicing Variant in a Case of Infantile Parkinsonism–Dystonia Type 2

**DOI:** 10.1155/crnm/2230320

**Published:** 2026-08-25

**Authors:** Ali Nikkhah, Reza Shervin Badv, Seyed Amir Hasan Habibi, Giovanna Zorzi, Maryam Mehdizadeh

**Affiliations:** ^1^ Department of Pediatric Neurology, Pediatrics Center of Excellence, Children’s Medical Center, Tehran University of Medical Sciences, Tehran, Iran, tums.ac.ir; ^2^ Pediatric Division, Vali-e Asr Hospital, Imam Khomeini Hospital Complex, Tehran University of Medical Sciences, Tehran, Iran, tums.ac.ir; ^3^ Department of Neurology, School of Medicine, Hazrat-e Rasool General Hospital, Iran University of Medical Sciences, Tehran, Iran, iums.ac.ir; ^4^ Department of Child Neurology, Fondazione IRCCS Istituto Neurologico Carlo Besta, Milan, Italy, istituto-besta.it; ^5^ Geriatric Mental Health Research Center, School of Behavioral Sciences and Mental Health (Tehran Institute of Psychiatry), Iran University of Medical Sciences, Tehran, Iran, iums.ac.ir

**Keywords:** dystonia, infant, movement disorders, Parkinsonism, *SLC18A2*

## Abstract

Parkinsonism–dystonia Type 2 (PKDYS2) is a rare autosomal recessive disorder caused by *SLC18A2* variants affecting the vesicular monoamine transporter 2 (VMAT2). We report the first genetically confirmed Iranian patient, an 18‐month‐old boy presenting with profound developmental delay, axial hypotonia, limb hypertonia, dystonia, oculogyric crises, ptosis, and autonomic dysfunction. Brain MRI, metabolic studies, and neurophysiological evaluations were normal. Whole‐exome sequencing identified a novel homozygous canonical splice‐site variant in *SLC18A2* (NM_003054.6:c.1071‐2A > G), confirmed by Sanger sequencing and absent from population databases. SpliceAI predicted loss of the native splice acceptor site (DS_AL = 0.74), supporting a deleterious effect on RNA splicing. According to ACMG criteria, the variant was classified as likely pathogenic. Levodopa–benserazide failed to provide sustained benefit and caused irritability and insomnia, whereas pramipexole produced modest improvement in bradykinesia, ptosis, and sweating with persistence of dystonia and oculogyric crises. Comparison with reported international cases shows a consistent phenotype and limited therapeutic response. This case expands the mutational spectrum of *SLC18A2*‐related disease and highlights the importance of early genetic diagnosis.

## 1. Introduction

Infantile parkinsonism–dystonia Type 2 (PKDYS2) is a rare autosomal recessive disorder caused by loss‐of‐function mutations in *SLC18A2*, which encodes the vesicular monoamine transporter 2 (VMAT2) [1, 2]. VMAT2 is an ATP‐dependent transmembrane protein critical for packaging dopamine, serotonin, norepinephrine, and other monoamines into synaptic vesicles [1, 2]. Dysfunction of VMAT2 leads to impaired monoaminergic neurotransmission, producing a constellation of neurologic features. First described in 2013 in a large consanguineous Saudi family (p.Pro387Leu founder mutation) [2], PKDYS2 has now been reported in dozens of patients worldwide [1, 3]. Common clinical findings include global developmental delay, axial hypotonia, limb rigidity/spasticity, parkinsonism with dystonia, and episodic oculogyric crises. Autonomic dysfunction (such as sweating, temperature instability, hypersalivation, and gastrointestinal dysmotility) is a frequent hallmark [1]. Notably, routine cerebrospinal fluid neurotransmitter levels (HVA, 5‐HIAA) are usually normal in PKDYS2, distinguishing it from other monoamine disorders [3].

Therapeutically, PKDYS2 has proved challenging. In contrast to classic infantile dopamine‐deficiency syndromes, levodopa generally fails to improve and often aggravates movement symptoms. By contrast, dopamine agonists (particularly pramipexole) have yielded partial benefit in some patients, especially those with the p.Pro387Leu variant [3]. A recent large cohort study (42 patients) confirmed that dopamine agonists produced only mild, often transient improvement in most treated individuals [1]. This underscores the need for supportive and individualized management in PKDYS2.

We report the first genetically confirmed Iranian patient with PKDYS2, carrying a novel *SLC18A2* splice‐site mutation. To place the present case in the context of the existing literature, we additionally summarized the reported genotypic and phenotypic characteristics of previously published PKDYS2 cases (Table 1). This comparison facilitates assessment of the clinical similarities and differences among affected individuals and highlights the expanding mutational spectrum of *SLC18A2*‐related disease. This case highlights the typical clinical syndrome, response to therapy, and the importance of genetic diagnosis for guiding care.

**TABLE 1:** Summary of reported *SLC18A2* variants and clinical features in PKDYS2.

Study	Variant(s)	Number of patients	Major clinical features	Response to levodopa	Response to dopamine agonists	Outcome
Rilstone et al. [4]	p.Pro387Leu	5	Hypotonia, dystonia, parkinsonism, oculogyric crises	Poor/Worsened	Partial improvement	Variable
Jacobsen et al. [5]	p.Pro387Leu	1	Severe infantile parkinsonism–dystonia	Poor	Partial improvement	Survived
Padmakumar et al. [6]	Missense variant	1	Developmental delay, hypotonia, dystonia	Poor	Limited benefit	Survived
Zhai et al. [7]	p.Pro237His	1	Severe hypotonia, dystonia, tremor	Poor	Mild improvement	Severe disease
Saida et al. [1]	Multiple variants	42	Developmental delay, hypotonia, dystonia, autonomic dysfunction, oculogyric crises	Mostly ineffective/worsened	Mild benefit in many patients	Genotype‐dependent
Almuqbil et al. [2]	p.Pro387Leu	3	Typical PKDYS2 phenotype	Poor	Partial improvement	Variable
Kaasalainen et al. [3]	Novel variant	1	Infantile dystonia–parkinsonism	Poor	Partial improvement	Survived
Janardhanan [8]	Novel genotype	1	Infantile parkinsonism–dystonia	Poor	Partial improvement	Ongoing
Present case	c.1071‐2A > G (splice‐site)	1	Developmental delay, hypotonia, dystonia, oculogyric crises, autonomic dysfunction	Ineffective; worsened irritability and insomnia	Modest improvement	Alive at 18 months

## 2. Case Presentation

**FIGURE 1:**
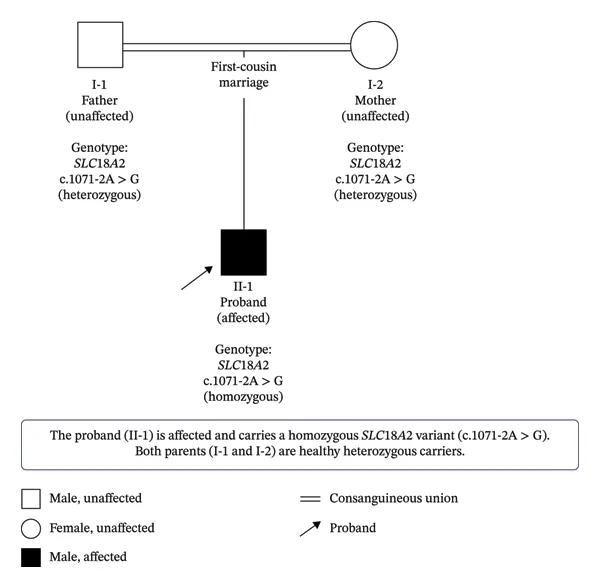
Pedigree of the family. The pedigree shows an autosomal recessive inheritance pattern.

An 18‐month‐old Iranian boy, born at term to healthy first‐cousin parents, presented with profound neurodevelopmental impairment. There was no history of neurological or movement disorders among first‐ or second‐degree relatives. A pedigree illustrating the family structure is provided in Figure 1. Whole‐exome sequencing (WES) identified a homozygous *SLC18A2* splice‐site variant in the proband, and Sanger sequencing confirmed that both unaffected parents were heterozygous carriers of the same variant. Together with the consanguineous parental relationship, these segregation findings support an autosomal recessive mode of inheritance. He had never achieved head control and exhibited marked axial hypotonia with hypertonicity of the limbs. Physical examination also revealed mild bilateral ptosis and preserved visual tracking. There was no history of neonatal complications or dysmorphic features. By 6 months of age, head lag and poor truncal tone were evident. Early investigations were unremarkable: Brain MRI and routine metabolic screens (including amino acids, urine organic acids, lactate, and ammonia) were normal. Evoked potentials (ABR and VEP) and electrodiagnostic studies (nerve conduction and EMG) showed no abnormalities.

Between 8 and 10 months, the patient developed multiple daily episodes of generalized dystonic posturing accompanied by sustained upward deviation of the eyes (oculogyric crises) with preserved awareness. These events were initially misinterpreted as seizures, and he received levetiracetam and primidone without benefit. Video‐EEG captured one event and demonstrated no epileptiform activity, confirming a nonepileptic movement disorder. By 10–14 months, developmental progress plateaued, and motor symptoms worsened. He also developed autonomic symptoms, including unexplained profuse sweating and nocturnal irritability with insomnia.

Given the constellation of hypotonia, dystonia with eye deviation, and autonomic features, an inherited monoamine disorder was suspected. Analysis of cerebrospinal fluid neurotransmitters was discussed but not performed due to parental reluctance. WES was performed using a clinical‐grade next‐generation sequencing platform. The mean sequencing depth across targeted regions was 100×, with 95.4% of target regions covered at ≥ 50×. Raw sequencing data were processed using a standard bioinformatics pipeline, including alignment to the human reference genome (GRCh38), variant calling, and annotation using validated clinical databases. Variants were filtered based on allele frequency, predicted functional impact, and autosomal recessive inheritance pattern. All protein‐coding regions of known disease‐associated genes were analyzed.

WES identified a homozygous splice‐site variant in *SLC18A2* (NM_003054.6:c.1071‐2A > G). The variant was confirmed by Sanger sequencing in both parents in a heterozygous state, consistent with autosomal recessive inheritance. The variant is absent from population databases (gnomAD and others) and has not been previously reported. In silico splice prediction using SpliceAI predicted loss of the native splice acceptor site (DS_AL = 0.74), supporting a deleterious effect on normal RNA splicing. No other pathogenic or likely pathogenic variants were identified that could explain the phenotype.

According to ACMG guidelines, the variant was classified as likely pathogenic based on its location at a canonical splice acceptor site in a gene in which loss‐of‐function is an established disease mechanism (PVS1), its absence from population databases (PM2), and the highly specific clinical phenotype consistent with *SLC18A2*‐related infantile parkinsonism–dystonia (PP4). The SpliceAI prediction further supported disruption of normal splicing. Serum copper and ceruloplasmin were normal, excluding Menkes disease.

The patient’s management focused on dopaminergic therapy and supportive care. A brief timeline of treatment response is provided to clarify the sequence and temporal evolution of clinical changes following initiation and modification of dopaminergic therapy. Levodopa–benserazide (Madopar) was initiated at 2 mg/kg/day and rapidly titrated to 5 mg/kg/day over ten days. Initially, a slight gain in head control was observed. However, after 1 month, the family noted increased irritability, hyperactivity, and difficulty sleeping. Because these adverse effects outweighed any benefit, levodopa was discontinued. Treatment was switched to the dopamine agonist pramipexole at 0.005 mg/kg/day (divided TID), subsequently increased to 0.01 mg/kg/day. Over several weeks, this produced modest improvement: The child had less bradykinesia, and autonomic signs (excessive sweating, ptosis) eased somewhat. However, severe axial hypotonia, limb rigidity, and the characteristic oculogyric crises persisted. No new adverse effects were reported with pramipexole at these low doses. The patient continues on pramipexole with ongoing occupational therapy and multidisciplinary rehabilitation.

## 3. Discussion

PKDYS2 is an exceedingly rare disease affecting the neurotransmitter system. Its phenotype reflects the loss of VMAT2‐mediated vesicular storage of monoamines, resulting in the depletion of dopamine, serotonin, and other biogenic amines at synapses [1, 2]. Consistent with previous reports, our patient exhibited a combination of global developmental delay, axial hypotonia, limb hypertonia/dystonia, and oculogyric crises [1]. As summarized in Table 1, the clinical manifestations of our patient largely overlap with the core phenotype reported across previously published PKDYS2 cases, supporting the phenotypic consistency of *SLC18A2*‐related disease despite considerable genetic heterogeneity. Autonomic disturbance (profuse sweating) was prominent, aligning with “treatable neurotransmitter” features described in the literature [2]. Unlike some cases, he never had clinical seizures, and his MRI brain remained normal—again reflecting the variability seen in reported cohorts (indeed, many PKDYS2 patients have unremarkable neuroimaging) [1].

The genetic finding of a novel homozygous splice‐site variant in *SLC18A2* (c.1071‐2A > G) expands the mutational spectrum of PKDYS2. Although functional RNA studies were not performed, the variant affects a canonical splice acceptor site and SpliceAI predicted loss of the native splice acceptor site (DS_AL = 0.74), supporting a loss‐of‐function effect. No other diagnoses (metabolic or genetic) explained this clinical picture. Splice‐site and missense mutations in *SLC18A2* have now been reported worldwide—to date, > 50 patients in ∼40 families [1, 3]. Genotype–phenotype correlations are emerging: Notably, the p.Pro237His variant (as in Zhai et al., who reported an infantile case) is associated with a more severe course and higher mortality, whereas p.Pro387Leu often has a somewhat milder phenotype [1, 7]. In Saida et al.’s series, 9 of 17 patients with Pro237His died by age 13 years, whereas all 14 patients with Pro387Leu survived [1]. Our novel variant’s prognosis is unknown, but his relatively stable status at 18 months is clinically reassuring, although long‐term prognosis remains uncertain.

Therapeutically, our experience mirrored published findings. The paradoxical worsening observed with levodopa may be explained by VMAT2 dysfunction, which impairs vesicular monoamine storage and disrupts synaptic dopamine handling. In this context, exogenous levodopa may lead to increased cytosolic dopamine without adequate vesicular sequestration, potentially contributing to neurochemical imbalance and symptom exacerbation. In addition, postsynaptic dopamine receptor supersensitivity secondary to chronic monoaminergic deficiency may contribute to the adverse response. In contrast, dopamine agonists such as pramipexole may provide partial symptomatic benefit by directly stimulating postsynaptic dopamine receptors and partially bypassing presynaptic vesicular dysfunction. Although a transient improvement in head control was initially observed, no sustained clinical benefit was achieved with levodopa, and treatment was associated with irritability and insomnia. This adverse response is well‐documented: Levodopa has generally shown limited efficacy and may worsen symptoms in many reported patients [1]. In contrast, we noted a partial response to a dopamine agonist. Pramipexole led to transient gains in head control, swallowing, and reduced autonomic signs, albeit with persistence of dystonia. Similar modest improvements on dopamine agonists have been reported. For example, Zhai et al. treated an infant with p.Pro237His with pramipexole and noted a slight reduction of a static tremor [7]. In the large Genet Med cohort, 18 of 21 patients treated with pramipexole showed at least mild improvement [1, 3, 6, 9, 10]. Conversely, further dose escalation often provokes side effects (as also seen in the Saudi series and in Kaasalainen et al.’s Finnish case) [2, 3]. Thus, dopamine agonists may be tried but generally achieve only partial, short‐lived benefit [1–3, 5, 8]. No other targeted therapies (e.g., serotonin precursors and VMAT2 gene therapy) are available at present.

This case underscores the critical role of genetic diagnosis. Beyond being the first report from Iran, this case introduces a novel *SLC18A2* splice‐site variant (c.1071‐2A > G) not previously described in any population database, further expanding the mutational spectrum of PKDYS2. The clinical phenotype remains highly consistent across reported cases, supporting the concept of a relatively stable core phenotype despite underlying genetic heterogeneity. The phenotypic overlap of PKDYS2 with more common conditions (e.g., epilepsy or cerebral palsy) can lead to misdiagnosis and inappropriate treatment [2, 3, 9]. Prompt genetic testing in infants with unexplained neuroregression and movement disorder can rapidly clarify the diagnosis, avoid unnecessary interventions, and guide management. As demonstrated by PKDYS2 and other neurotransmitter disorders, accurate molecular diagnosis enables tailored therapy: For instance, it steers clinicians away from futile levodopa trials and toward cautious dopamine‐agonist use [1–10].

A limitation of this report is the absence of standardized quantitative rating scales for assessing the severity of dystonia and parkinsonism before and after treatment, which limits objective comparison of therapeutic response. In addition, pre‐ and posttreatment video recordings were not available due to practical constraints in the clinical setting. Consequently, assessment of treatment response was based on longitudinal clinical observation and neurological examination. Future studies should incorporate validated movement disorder rating scales and systematic video documentation to enable more objective and reproducible evaluation of therapeutic outcomes in patients with *SLC18A2*‐related disorders.

We describe the first Iranian infant diagnosed with PKDYS2, harboring a novel *SLC18A2* splice‐site variant. His clinical presentation—developmental delay, hypotonia, parkinsonism–dystonia, oculogyric crises, and autonomic symptoms—aligns closely with the expanding PKDYS2 phenotype. The case reinforces several key points: (1) *SLC18A2*‐related PKDYS2 should be considered in infants with unexplained hypotonia and dystonia, especially in consanguineous populations; (2) early genetic testing expedites diagnosis and management; (3) levodopa is generally ineffective and may worsen symptoms in PKDYS2. This report adds to the global literature on PKDYS2 and highlights the necessity of individualized care based on genotype–phenotype correlations.

## Author Contributions

Dr. Ali Nikkhah and Dr. Reza Shervin Badv collected clinical data and drafted the initial manuscript.

Dr. Ali Nikkhah and Dr. Seyed Amir Hasan Habibi conducted literature review and contributed to the discussion and interpretation of findings.

Dr. Giovanna Zorzi and Dr. Seyed Amir Hasan Habibi supervised genetic analysis, and reviewed and revised the manuscript critically for important intellectual content.

Dr. Maryam Mehdizadeh and Dr. Seyed Amir Hasan Habibi conceived and designed the report, provided overall supervision, and finalized the manuscript for submission.

## Funding

No specific funding was received for this work.

## Disclosure

All authors read and approved the final version of the manuscript.

## Ethics Statement

Informed patient consent was obtained from the patient’s parents for the publication of clinical details and genetic results, which was approved by Tehran University of Medical Sciences’ ethics committee. We confirm that we have read the Journal’s position on issues involved in ethical publication and affirm that this work is consistent with those guidelines.

## Consent

All the patients allowed personal data processing, and informed consent was obtained from all individual participants included in the study.

## Conflicts of Interest

The authors declare no conflicts of interest.

## References

[1] Saida K. , Maroofian R. , Sengoku T. et al., Brain Monoamine Vesicular Transport Disease Caused by Homozygous SLC18A2 Variants: a Study in 42 Affected Individuals, Genetics in Medicine. (2023) 25, no. 1, 90–102, 10.1016/j.gim.2022.09.010. 36318270

[2] Almuqbil M. A. , Tabassum S. , Muthaffar O. Y. et al., Parkinsonism‐dystonia‐2: Case‐Series Study from Saudi Arabia, Annals of Clinical and Translational Neurology. (2024) 11, no. 4, 1063–1066, 10.1002/acn3.52020. 38389300 PMC11021621

[3] Kaasalainen S. , Arikka H. , Martikainen M. H. , and Kaasinen V. , Novel SLC18A2 Variant in Infantile Dystonia‐Parkinsonism Type 2, Case Reports in Neurological Medicine. (2024) 2024, no. 1, 10.1155/2024/4767647. PMC11074866 38716424

[4] Rilstone J. J. , Alkhater R. A. , and Minassian B. A. , Brain dopamine–serotonin Vesicular Transport Disease and Its Treatment, New England Journal of Medicine. (2013) 368, no. 6, 543–550, 10.1056/nejmoa1207281. 23363473

[5] Jacobsen J. C. , Wilson C. , Cunningham V. et al., Brain dopamine-serotonin Vesicular Transport Disease Presenting as a Severe Infantile Hypotonic Parkinsonian Disorder, Journal of Inherited Metabolic Disease. (2016) 39, no. 2, 305–308, 10.1007/s10545-015-9897-6. 26497564

[6] Padmakumar M. , Jaeken J. , Ramaekers V. et al., A Novel Missense Variant in SLC18A2 Causes Recessive Brain Monoamine Vesicular Transport Disease and Absent Serotonin in Platelets, JIMD reports. (2019) 47, no. 1, 9–16, 10.1002/jmd2.12030. 31240161 PMC6498820

[7] Zhai H. , Zheng Y. , He Y. et al., A Case Report of Infantile parkinsonism-dystonia-2 Caused by Homozygous Mutation in the SLC18A2 Gene, International Journal of Neuroscience. (2023) 133, no. 5, 574–577, 10.1080/00207454.2021.1938036. 34078222

[8] Janardhanan A. , SLC18A2 Associated infantile-onset-parkinsonism-dystonia 2: a Novel Genotype, Parkinsonism & Related Disorders. (2025) . 10.1016/j.parkreldis.2025.107930 40543173

[9] Kurian M. A. , Gissen P. , Smith M. , Heales S. J. , and Clayton P. T. , The Monoamine Neurotransmitter Disorders: an Expanding Range of Neurological Syndromes, The Lancet Neurology. (2011) 10, no. 8, 721–733, 10.1016/s1474-4422(11)70141-7. 21777827

[10] Lohr K. M. and Miller G. W. , VMAT2 and Parkinson’s Disease: Harnessing the Dopamine Vesicle, 2014, Taylor & Francis, 1115–1117. 10.1586/14737175.2014.960399 PMC4479119 25220836

